# High Genetic Similarity of MRSA ST88 Isolated From Pigs and Humans in Kogi State, Nigeria

**DOI:** 10.3389/fmicb.2018.03098

**Published:** 2018-12-17

**Authors:** Otalu Jnr Otalu, Jacob K. P. Kwaga, Emmanuel Chukuwdi Okolocha, Md Zohorul Islam, Arshnee Moodley

**Affiliations:** ^1^Department of Microbiology, Faculty of Natural Sciences, Kogi State University, Anyigba, Nigeria; ^2^Department of Public Health and Preventive Medicine, Faculty of Veterinary Medicine, Ahmadu Bello University, Zaria, Nigeria; ^3^Department of Veterinary and Animal Sciences, Faculty of Health and Medical Sciences, University of Copenhagen, Copenhagen, Denmark

**Keywords:** CA-MRSA, porcine, ST88, staphylococci, zoonotic

## Abstract

We determined the prevalence and genetic characteristics of methicillin-resistant *Staphylococcus aureus* (MRSA) isolated from pigs and humans between September 2013 and February 2015 in Kogi State, a central region in Nigeria. A total of 680 nasal swabs were collected and analyzed from pigs (*n* = 425) and “pig-contact” humans (*n* = 55) on 35 farms, and “non-pig-contact” humans (*n* = 200). MRSA was recovered from 20 (4.7%) pigs on 12 farms and 18 (7.0%) humans. Six (2.4%) of the human isolates were recovered from “pig-contact” humans, of which only three work on farms also harboring MRSA positive pigs. All 38 MRSA were resistant to β-lactams only, belonged to *spa* type t1603, sequence type (ST) 88, and *mec*A was associated with a SCC*mec* IVa element. Four isolates from a pig, a pig-contact human from the same farm, a pig-contact human from a pig farm in a different district, and a non-pig-contact human were subjected to whole genome sequencing (WGS). Core genome SNP analysis revealed high genetic similarity between strains (3–11 SNP differences), despite the temporal (2 year gap) and geographic (165 km) differences between isolates. Furthermore, these Nigerian isolates form a distinct clade when compared to other African MRSA ST88 isolates. All but one porcine strain was positive for *scn* suggesting a possible human origin and that pigs were either transiently contaminated by humans or result of a very recent human-to-pig transmission event. To our knowledge, this is the first report of genetically confirmed MRSA in pigs in Nigeria, which appear to be a typical CA-MRSA clone present in the human population.

## Introduction

Methicillin-resistant *Staphylococcus aureus* (MRSA) is of great concern in both human and veterinary medicine (Vanderhaeghen et al., 2010). Such concerns are related to difficulty in treating infections, prolonged hospitalization and increased health care costs. Pigs can be asymptomatic carriers of *S. aureus* including methicillin-resistant strains, although infections with *S. aureus* are infrequent (van der Wolf et al., 2012). The central concern with MRSA in pigs is the risk of spread to humans (Unnerstad et al., 2017).

MRSA sequence type (ST) 398 is the predominant clone observed in pigs from Europe and North America, whereas MRSA ST9 is predominant in Asia. In Africa, there are only four reports of MRSA in pigs; two in South Africa (Adegoke and Okoh, 2014; Van Lochem et al., 2018), one in Senegal (Fall et al., 2012), and one in Nigeria (Okunlola and Ayandele, 2015). Unfortunately, only two of the four studies used molecular methods to confirm the *S. aureus* species identification and the presence of *mecA* in oxacillin or cefoxitin resistant strains, and only one study further typed the MRSA strains. Fall et al. (2012) reported that their porcine MRSA belonged mainly to ST5 and a singleton ST88, which are both major MRSA lineages responsible for human infections in Senegal (Breurec et al., 2011). Pigs have been shown to harbor human-associated MRSA clones e.g., USA300 (Arriola et al., 2011; Baez et al., 2017). People in close contact with pigs (farmers, veterinarians, transporters, and slaughterhouse workers) have been shown to be at a greater risk of being colonized with livestock associated MRSA (LA-MRSA) (Denis et al., 2009; Liu et al., 2015). However, LA-MRSA has been reported in people with no known contact to pigs or pig farms (Larsen et al., 2017). In Nigeria, there are no published data regarding colonization of pigs with MRSA and possible transmission of strains between pigs and humans. Our objective was to evaluate the occurrence of MRSA in pigs, and compare the strains to those isolated from pig-contact and non-contact humans.

## Materials and Methods

### Ethical Statement

The study and protocol were approved by the institutional review board of Kogi State University Teaching Hospital. Informed oral consent was obtained from each participant and pig farm owners consented to the sampling of their animals.

### Sample Collection

**Figure 1:**
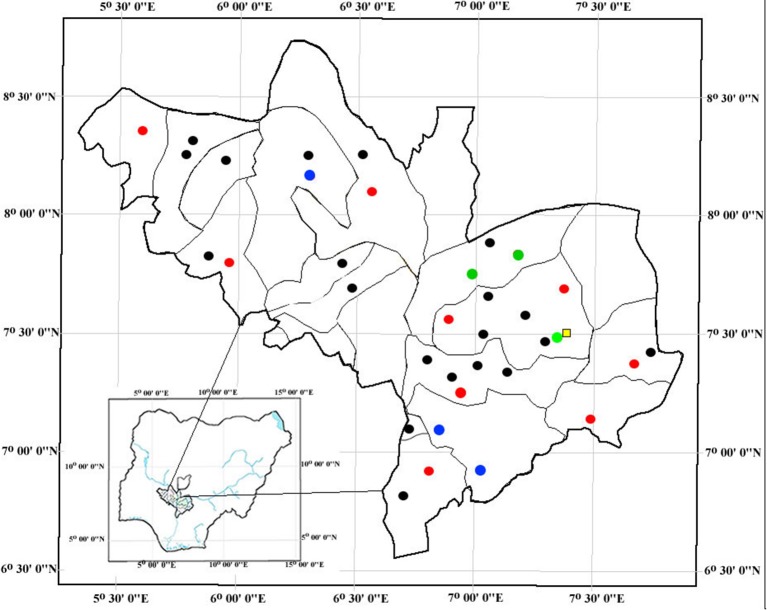
Geographic location of pig farms in the 21 local government areas and Kogi State University (represented by a yellow square). Black dots indicate MRSA negative pig farms. Red dots indicate MRSA positive pig farm where only pigs were positive. Blue dots indicate pig farms where only humans were positive and no pigs. Green dots indicate pig farms harboring both MRSA positive pigs and humans.

The study was conducted between September 2013 and February 2015 and covered 16/21 local government areas of Kogi State (Figure 1). Samples were collected from 35 pig farms housing between 28 and 275 pigs per farm. Between 10 and 15 pigs were randomly sampled per farm. When pigs were housed in pens (i.e., on 5/35 farms), two pigs per pen were sampled. On those farms not housing pigs in pens, pigs were randomly selected. Human samples were collected from 55 pig farms workers (pig contact humans). The number of farm workers per farm ranged from 1 to 6 individuals (mode = 1). Two hundred students attending Kogi State University in Anyigba that had no pig contact were also sampled (non-pig-contact humans). All human participants were provided with a standardized questionnaire to collect demographic data, medical history over the previous 12 months e.g., hospitalization and antimicrobial therapy, and information about contact with pigs.

### Isolation and Characterization of MRSA

Nasal swabs were collected with a sterile cotton-tip swab inserting into both anterior nares of each pig and human and were placed into 3 ml Mueller Hinton broth (Oxoid, UK) supplemented with 6.5% sodium chloride and incubated at 37°C for 24h. A loopful of the inoculum from the enrichment broth was streaked on Brilliance agar (Oxoid, UK). After incubation at 37°C for 24h, suspected MRSA colonies (denim blue colonies) were sub-cultured on 5% sheep blood agar and incubated at 37°C for 24h. Isolates were speciated by matrix-assisted laser desorption/ionization time-of-flight mass spectrophotometry (MALDI-TOF MS) (BioMérieux, France). Confirmed *S. aureus* isolates were stored at −20°C for further analysis. Antimicrobial susceptibility testing was done using the Sensititre system and COMPAN1F MIC plate (ThermoFisher Scientific, USA), and susceptibility was interpreted according to CLSI (Clinical and Laboratory Standards Institute (CLSI), 2014). MRSA were characterized by a multiplex PCR to detect *spa, mec*A, *lukF-PV* (a marker of the Panton-Valentine leucocidin, PVL)*, scn* and a CC398 specific band (Islam et al., 2017). Direct sequencing of the purified PCR reaction was used for *spa* typing (Harmsen et al., 2003). Four isolates (one pig, one pig-contact human from the same pig farm, one non-pig-contact human from the same district, and one pig-contact human from a different district) were selected for further SCC*mec* (Kondo et al., 2007), multi locus sequencing typing (Enright et al., 2000), and whole genome sequencing (WGS).

### Whole-Genome Sequencing

Overnight cultures were grown in tryptic soy broth at 37°C with 200-rpm shaking. Genomic DNA was extracted from cultures by using the automated Maxwell DNA instrument using the Maxwell® RSC Cultured Cells DNA Kit (Promega, United Kingdom). Library preparation was carried out using the Nextera XT kit and paired end 2 × 250 bp sequencing on the MiSeq, all following standard Illumina protocols (Illumina, Inc., United States). All raw reads have been deposited in the Sequence Read Archive database in the European Nucleotide Archive under the study accession number PRJEB26533.

### Phylogenetics and Comparative Genomics

All analyses were performed in CLC Genomics Workbench v. 11.0 using the tools within the Microbial Genomics Module. First, Kmer analysis was performed against the NCBI RefSeq database and including only complete staphylococcal genomes. This analysis was performed to determine if there was any contamination of the DNA prior to sequencing and as a secondary confirmation of the *S. aureus* species identification. Secondly, Adapter trimmed, high quality reads were mapped against the reference genome AUS0325, a closed ST88 methicillin-susceptible, penicillin-resistant genome (NCBI accession no. LT615218) to identify single nucleotide polymorphisms (SNPs). A maximum likelihood tree was generated from core genome SNPs. To further investigate the clonal relationship of MRSA ST88 from the African continent, our strains were compared to the 17 publically available ST88 MRSA isolated in Ghana (Kpeli et al., 2017). The Resfinder v.3.0 and Virulence finder v.1.5 web-based pipelines were used to search for the presence of known virulence and antibiotic resistance genes (Zankari et al., 2012; Joensen et al., 2014).

## Result

MRSA was recovered from 20/425 (4.7%) pigs, 6/55 (10.9%) pig farms workers, and 12/200 (6.0%) non-pig contact humans (Table 1). The MRSA positive pigs came from 12 farms (Figure 1). Despite sampling multiple pigs per farm, only 4/12 farms had >1 MRSA positive pigs. Six of the human MRSA isolates were recovered from “pig contact” humans on six pig farms. However, only 3/6 individuals came from farms, which also harbored MRSA positive pigs. Moreover, despite sampling more than one pig farm worker on these six farms, only one individual per farm was positive. The remaining 12 MRSA were isolated from University students not having any pig contact. None of the MRSA positive individuals had reported taking antibiotics and only one pig farm worker had reported being hospitalized in the last 12 months prior to sampling.

**Table 1:** Characterization of MRSA isolated from pigs, pig farm workers and non-pig contact humans.

	**Number of sampled pigs/humans**	**Number of pig farms**	**Number of MRSA positive farms**	**Number of MRSA positive samples**	**Location of MRSA positive farms/students**	**Sampling period**	**Presence of *scn* amongst MRSA isolates**
Pigs	425	35	12	20	Ankpa, Anyigba, Echeno, Egbe, Ejule, Itama, Lokoja, Ogidi, Oguma, Okpo, Okura-Offante, Sheria	Nov 2013–Jan 2015	19
Pig farm workers	55	35	6^*^	6	Ajaka, Anyigba, Elechi, Kabba, Oguma, Sheria	Nov 2013–Jan 2015	6
Kogi State University students	200	–	–	12	Anyigba	May–Nov 2014	12

* *3/6 farms harbored MRSA positive pigs. Underlined location of the farms*.

All 38 MRSA isolates were resistant to penicillin and cefoxitin only and there was agreement between phenotypic and inferred genotypic resistance. All strains were positive for the CC398 specific band. However, *spa* sequencing demonstrated that all strains had the same *spa* type, t1603, which is unrelated to *spa* types typically associated with CC398. Furthermore, MLST demonstrated that strains belong to ST88. *In silico* PCR using the CC398 specific primers, which target the C398-specific *sau1-hsdS1* variant, (*FP2sau1*: 5′GAGAATGATTTTGTTTATAACCCTAG3′ and CC398r1: 5′-CAGTATAAAGAGGTGACATGACCCCT-3′) and our *de novo* assembled contigs, showed that the primers bind 100% and produce the expected 106 bp amplicon. The primers bind to an open reading frame (ORF) with 99% nucleotide identity to *hsdS*, which is the sequence specificity gene of the Sau1 type I restriction-modification system (data not shown). *mecA* was harbored on an SCC*mec* type VIa cassette.

**Figure 2:**
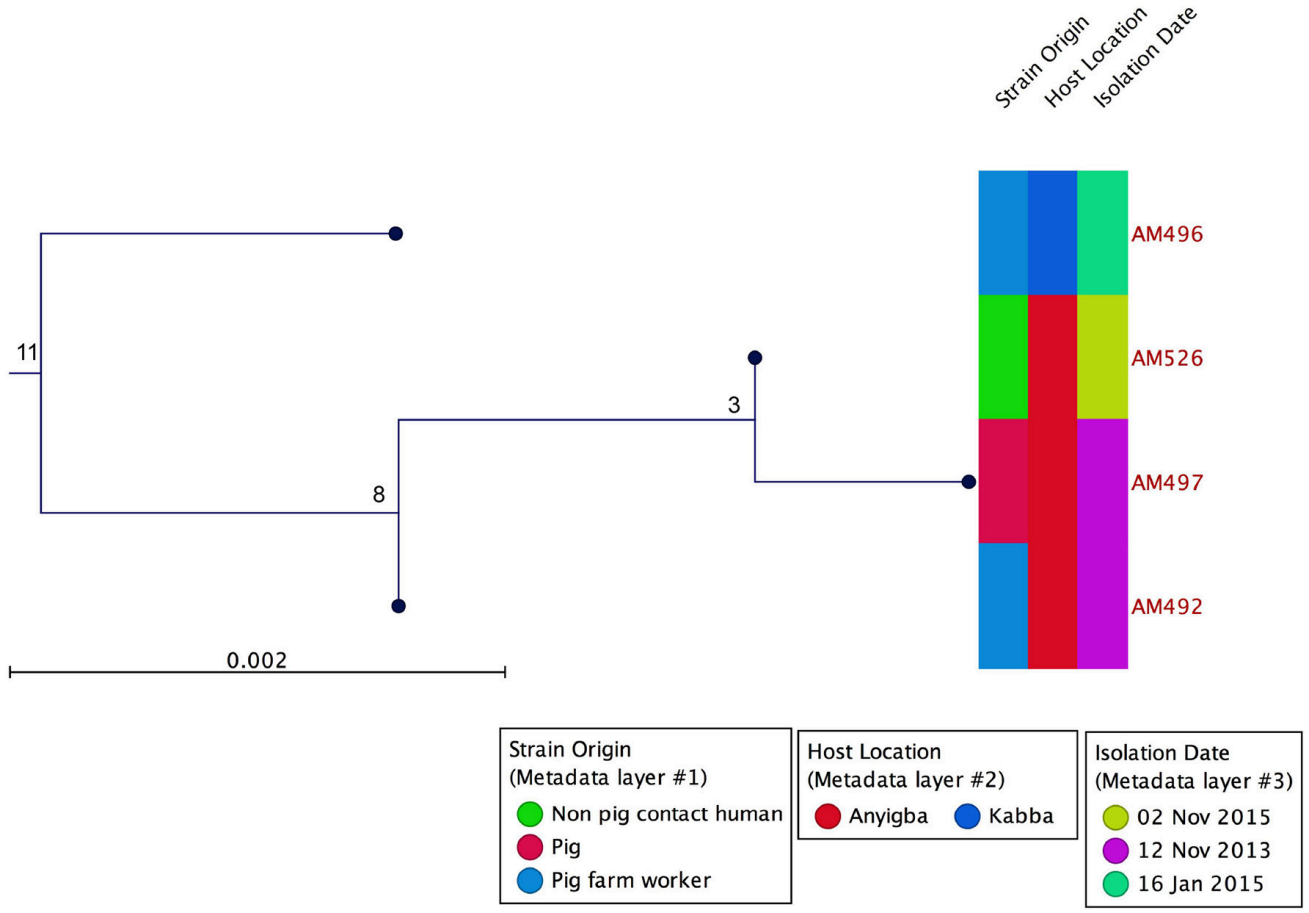
Core genome phylogeny using AUS0325 as the reference genome. MRSA ST88 from a pig, pig contact human and a non-pig contact human. Phylogeny based on an alignment of 1,248 SNPs. Metadata describing sample origins, sampling location in Kogi State and isolation date is provided. The numbers indicate SNP differences.

**Figure 3:**
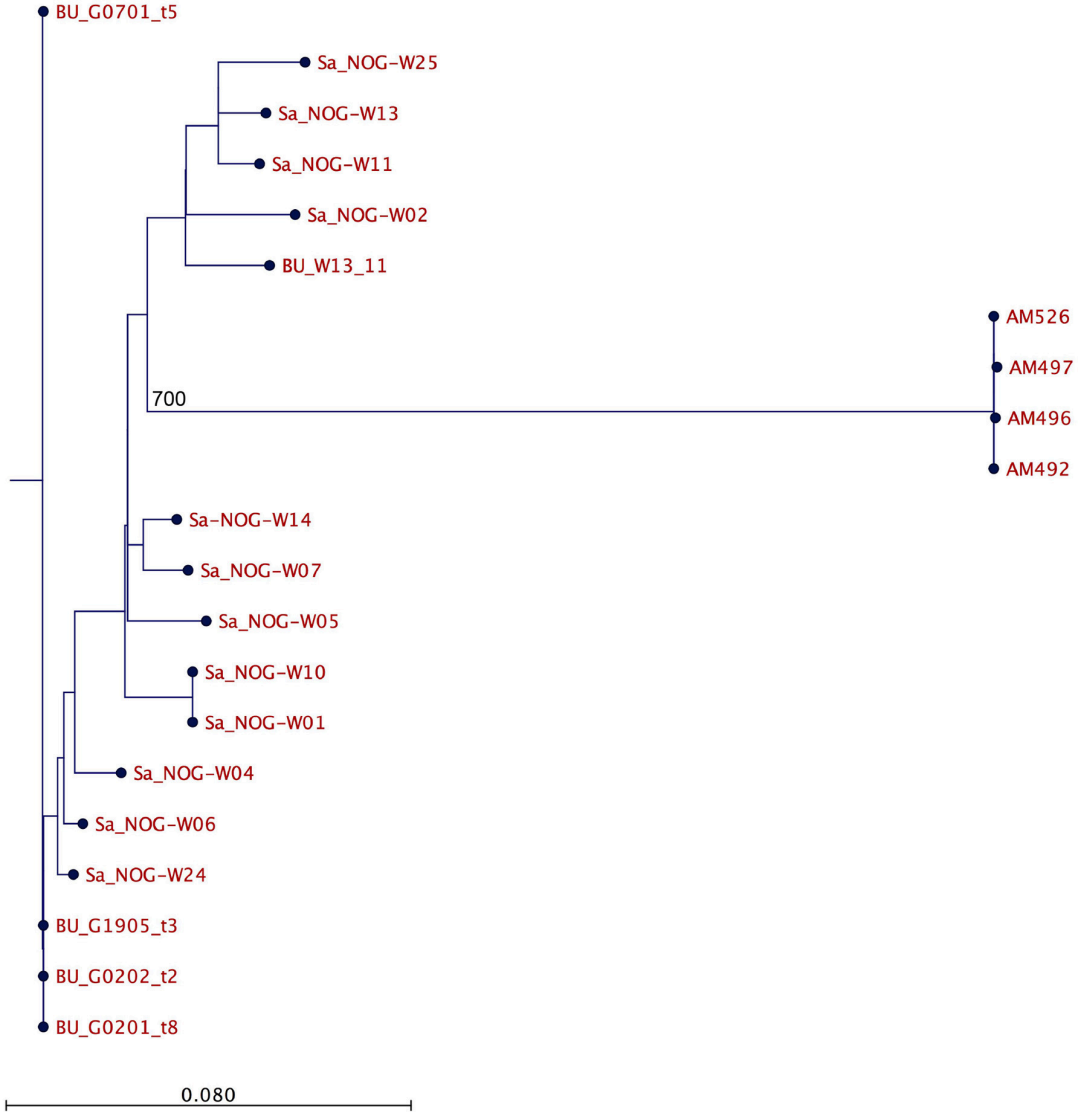
Comparative analysis of the Nigerian and Ghanaian MRSA ST88. Core genome phylogeny based on an alignment of 1492 SNPs. The number in the figure indicates SNP differences.

Based on WGS, the core genome consisted of 2407 coding DNA sequences (CDS) with *S. aureus* AUS0325 used as reference. A core genome phylogeny was inferred by mapping reads from the four sequenced isolates to AUS0325, and a phylogenetic tree was constructed using the core genome SNPs and maximum likelihood estimation. Relative to AUS0325, 1,248 SNPs were identified, but only 11 SNPs were found between the four isolates in this study despite the temporal and geographic differences (Table 2; Figure 2). Isolates from the same region i.e., Angyiba and despite a 2-year gap only differed by 3–8 SNPs. A phylogenetic comparison with the 17 ST88 from Ghana indicates that the Nigerian isolates formed a separate, distinct clade with 700 SNPs differences (Figure 3).

**Table 2:** Single nucleotide polymorphisms in AM492, AM496, AM497, and AM526 relative to the ST88 reference genome, AUS0325.

**Nucleotide position**	**AM526**	**AM497**	**AM492**	**AM496**
853428	T	T	C	C
853440	C	Gap	Gap	Gap
853452	C	Gap	Gap	Gap
1349949	T	G	T	T
2673340	G	G	G	A
2673364	A	A	A	G
2673388	G	G	Gap	Gap
2673400	G	G	Gap	Gap
2673412	Gap	Gap	Gap	G
2673424	Gap	Gap	G	G
2673436	Gap	Gap	G	G

All MRSA strains were *pvl* negative and 37/38 strains contained the human immune evasion gene *scn*. The only strain that was *scn-*negative was of porcine origin. Furthermore, based on analysis of the *de novo* contigs in Virulence Finder, all four strains contained the following putative virulence associated genes *scn, sak, lukE, lukD*, gamma hemolysin, *aur, splA*, and *splB*.

## Discussion

We describe for the first time the isolation of genetically confirmed MRSA in pigs and pig farm workers in Nigeria. This is only the fifth description of MRSA in healthy pigs on the African continent and appears not to be the typical livestock associated clones found in pigs worldwide namely CC398 and CC9.

All MRSA isolates found in this study belonged to ST88, which has been isolated from both hospital- and community- acquired infections in humans in several Sub-Saharan African countries, including Nigeria (Ghebremedhin et al., 2009; Shittu et al., 2012; Kolawole et al., 2013; Raji et al., 2013; Abdulgader et al., 2015). ST88 appears to be primarily associated with the African continent, but has also been reported in Asia and sporadically in other parts of the world (Schaumburg et al., 2014). The association of ST88 and livestock has only been described in Africa. Specifically, MRSA ST88 has been identified in one pig isolate from Senegal and healthy sheep in the Ivory Coast (Fall et al., 2012; Schaumburg et al., 2015). In China, MRSA ST88 has been found in food of animal origin and recently in livestock workers (Wang et al., 2014; Ye et al., 2016).

MRSA in pigs and pig farmers in Africa was first reported in Senegal (Fall et al., 2012) however, no MRSA was isolated from the farm workers. In Nigeria, there has been only one report of phenotypic oxacillin-resistant *S. aureus* in pigs from the Oyo State in South Western, Nigeria (Okunlola and Ayandele, 2015). Only 18/200 (9%) pigs were positive for presumptive MRSA on 7 of the 11 farms sampled. MRSA has been isolated from other animals in Nigeria namely; camels, sheep, cattle, goats and recently in poultry (Mai-siyama et al., 2014; Nworie et al., 2017). Similar to (Okunlola and Ayandele, 2015), we observed few MRSA positive animals on each farm. Moreover, all our MRSA isolates in this study belong to the same *spa* type (t1603) irrespective of the source (pig, pig-contact human or non-pig-contact human) and based on WGS the high genetic similarity, raises questions about the origin of these strains. The immune evasion cluster (IEC), which is carried on a bacteriophage and encodes the secreted proteins staphylococcal complement inhibitor (*scn*), staphylokinase (*sak*), and chemotaxis inhibitory protein (*chp*), are thought to contribute to the immune evasion in humans (van Wamel et al., 2006). Moreover, these genes are less prevalent in livestock adapted *S. aureus* lineages and are hence considered good genetic markers for identification of human associated *S. aureus* clones (McCarthy et al., 2011, 2012; Uhlemann et al., 2012). We found a high incidence of *scn* amongst our porcine isolates (19/20 isolates) and the additional occurrence of *sak* based on WGS data. The presence of these IEC related genes suggest a possible human origin and that pigs were either transiently contaminated by farm workers or the result of a very recent human-to-pig transmission event. Human associated *S. aureus* lineages have been previously described in animals including pigs (Arriola et al., 2011; Schaumburg et al., 2012; Nagel et al., 2013; Baez et al., 2017).

The MRSA ST88 in our study was only resistant to β-lactams. Typically, antibiotic resistance amongst animal *S. aureus* isolates tend be higher compared to human isolates. A study of methicillin resistant coagulase-negative staphylococci (MRCoNS) of porcine origin in Nigeria, found that isolates were multi-drug resistant (MDR), 85% of strains were resistant to fusidic acid, and harbored a number of different antibiotic resistance genes (Ugwu et al., 2015). CoNS are a reservoir of antibiotic resistance genes with the potential to transfer to *S. aureus*, as has been demonstrated with the plasmid-mediated transfer of *mupA*, conferring mupirocin resistance, from *S. haemolyticus* to *S. aureus* (Rossi et al., 2016). It is possible that these low-level resistant MRSA ST88 strains if allowed to adapt to pigs and occupy the same niche as MDR MRCoNS, provide an opportunity for the transfer of antibiotic resistance genes to potentially create MDR CA-MRSA.

All our strains were presumptively identified as belonging to CC398. However, upon additional strain typing, this was shown to be incorrect. Typing of MRSA is key for surveillance, epidemiological studies and infection control. Rapid, reliable identification methods such as PCR based methods are important tools to identify MRSA and type to the clonal complex level. The CC398 specific PCR is an excellent tool to rapidly identify *S. aureus* belonging to CC398, which was shown to be 100% specific for CC398 only (Stegger et al., 2011). This PCR is based on *sau1-hsdS* since it was previously shown that variations in this gene is associated with specific *S. aureus* clonal lineages but maintained a high sequence homology within the lineage (Waldron and Lindsay, 2006). This approach has also been used for rapid identification of hospital-acquired MRSA lineages (Cockfield et al., 2007). Unfortunately, at the time of the study by Stegger et al. (2011) there were no publically available ST88 genomes and strains belonging to ST88 were not included in the study since ST88 is the not the typical LA-MRSA and it is not a globally disseminated human associated clone. In light of our findings, we recommend that with the current PCR primers the CC398 specific PCR result should be complemented with *spa* typing or another method to verify the clonal lineage association.

Our study has some limitations. Ideally, all 38 MRSA strains should be whole genome sequenced to better understand ST88 strain diversity in this region and study possible transmission events. However, the latter would be difficult to assess as we do not have information about the movement of pigs e.g., via trade, and the contact and movement of people. Additionally, we could not sequence all strains because of financial limitations.

Livestock are known reservoirs of new pathogenic bacteria with the ability to cross the host species barrier, and evolve in a host-specific manner to become established in the human population. This has been demonstrated for LA-MRSA CC398 (Price et al., 2013). Further research is required in Nigeria to determine whether MRSA carriage amongst pigs was transient or persistent, establish the exact transmission routes on pig farms and explore biosecurity measures for preventing the spread of MRSA in the farm environment. While there are limitations to our study; cross-sectional study design conducted at one time point only, nonetheless we provide valuable information on a continent where there are limited studies describing the occurrence of MRSA in animals and even fewer providing molecular typing data.

## Author Contributions

OO, JK, and EO designed of the work. OO, JK, EO, MI, and AM performed the acquisition, the analysis, and interpretation of data. OO, JK, EO, MI, and AM drafted and revised the manuscript. All authors approved the final version of this manuscript.

### Conflict of Interest Statement

The authors declare that the research was conducted in the absence of any commercial or financial relationships that could be construed as a potential conflict of interest.
